# The Effects of Temperament and Character on Symptoms of Depression in a Chinese Nonclinical Population

**DOI:** 10.1155/2011/198591

**Published:** 2011-10-17

**Authors:** Zi Chen, Xi Lu, Toshinori Kitamura

**Affiliations:** ^1^Research Center of Applied Psychology, Chengdu Medical College, Chengdu 610083, China; ^2^Department of Applied Psychology, Chengdu Medical College, 601 Rongdu Road, Jinniu District, Chengdu 610083, China; ^3^Department of Clinical Behavioural Sciences (Psychological Medicine), Kumamoto University Graduate School of Medical Sciences, Kumamoto 860-8556, Japan; ^4^Kitamura Institute of Mental Health Tokyo, Tokyo, Japan

## Abstract

*Objective*. To examine the relations between personality traits and syndromes of depression in a nonclinical Chinese population. *Method*. We recruited 469 nonclinical participants in China. They completed the Chinese version temperament and character inventory (TCI) and self-rating depression scale (SDS). A structural equation model was used to rate the relation between seven TCI scales and the three SDS subscale scores (based on Shafer's meta-analysis of the SDS items factor analyses). This was based on the assumption that the three depression subscales would be predicted by the temperament and character subscales, whereas the character subscales would be predicted by the temperament subscales. *Results*. The positive symptoms scores were predicted by low self-directedness (SD), cooperativeness (C), reward dependence (RD), and persistence (P) as well as older age. The negative symptoms scores were predicted only by an older age. The somatic symptoms scores were predicted by high SD. *Conclusion*. Syndromes of depression are differentially associated with temperament and character patterns. It was mainly the positive symptoms scores that were predicted by the TCI scores. The effects of harm avoidance (HA) on the positive symptoms scores could be mediated by low SD and C.

## 1. Introduction

Depression is the most prevalent mental disorder in many countries. Personality has been extensively studied as a risky factor of depression. One of the most promising theories to understand depression from the personality perspective is Cloninger's biosocial personality model. This has come from behavioral genetics, neuropharmacology, and psychology, and it gives insight into the aetiology of depression [1–3]. This model posits seven personality traits: four temperament dimensions (novelty seeking (NS), harm avoidance (HA), reward dependence (RD), and persistence (P)) and three character dimensions (self-directedness (SD), cooperativeness (C), and self-transcendence (ST)). Temperament is determined by genetic structure and manifests itself as a heritable component of one's behaviour. It refers to reflective emotional reactions. Character refers to self-identity, which is acquired primarily through a socialisation process, although recent study also identified a hereditary contribution to the development of character. Character is considered to be evoked by temperament. Such interaction of the two dimensions enhances cognitive learning of an individual's self-concept throughout the lifespan [4].

There are many reports suggesting that high HA and low SD predict depression [5–12], although other subscales of temperament and character were found to be related to depression in a few studies. 

Almost all the studies on the association between personality traits and depression have been performed as if depression is a homogenous condition. However, factor analyses of depressive symptoms generally noted that depressive symptoms consisted of a few syndromes. Thus, a new paradigm may be requited from whether personality trait predicts depression to which personality traits predict which depressive syndrome. It should also be noted here that research has shown that the constructs of depressive symptoms in clinical and nonclinical populations are qualitatively identical [13]. Difference between clinical and nonclinical populations in depression is symptom severity [14, 15].

Another issue about the studies on the association between depression and personality—particularly temperament and character—is previous investigations treating temperament and character domains simultaneously predicting depression. However, Cloninger has posited that temperament is a set of reflect emotions on which character develops. Thus, it is feasible to speculate that the effects of temperament, if any, on depression may not be direct but be mediated via character. Hence, 0-order correlations between temperament subscale scores and depression scores may be spurious. This point has rarely been studied empirically.

The objective of this paper is to examine the relations between personality traits and syndromes of depression in a nonclinical Chinese population. We paid attention to the mediation of the effects of temperament on depression via character as well as differential association with depressive syndromes.

## 2. Methods

### 2.1. Participants and Procedure

The data of the present study came from a population of 486 inhabitants in Beijing City, Shenyang city, and Dalian city (all cities are located in the north eastern area of China). We distributed 500 set of questionnaires and stamped envelopes to office workers of three companies separately in above three cities. Usable questionnaires were returned by 469 participants. They were 235 men and 234 women. Their ages ranged between 18 and 81 years. Men were slightly but significantly (*t* = 1.98, *P* < 0.05) older (*M* = 42.8; SD = 11.7) than women (*M* = 40.6; SD = 11.9).

### 2.2. Measures

#### 2.2.1. Temperament and Character

The temperament and character inventory (TCI) [2] was used to assess two aspects of personality—temperament and character. Temperament, which is moderately heritable and stable throughout life, refers to automatic emotional responses to experiences. This includes four dimensions, NS, HA, RD, and P. Character refers to self-perception and individual differences in goals and values that influence voluntary choices, intentions, and the meaning of experiences throughout life. Character, which is also moderately heritable [16] but influenced by socio-cultural learning, matures in progressive steps throughout life. This factor includes SD, C, and ST. We used the 144-item Chinese version of the TCI [17]. Each scale of the TCI (NS, HA, RD, P, SD, C, and ST) consists of 20 items. Each item in the original version is rated with a 2-point scale (“yes” or “no”). In this study, items were rated using a 5-point scale (1 = “very unlikely” to 5 = “very likely”). This was because 5-point scales were more suitable for factor analysis compared with two-point scales.

#### 2.2.2. Depression

The self-rating depression scale (SDS) [18] was used to rate depressive mood. It consisted of 20 items selected by the factor analysis. It has been translated into a wide range of languages and its validity and reliability across cultures have been thoroughly assessed. From the time of the original report of the SDS, there have been efforts to evaluate factor structure of the SDS [19–21] and a number of factors structure models have been found.

### 2.3. Statistical Analysis

We followed the results of Shafer's [20] meta-analyses of SDS that confirmed three subscales—positive, negative, and somatic symptoms. The positive symptoms subscale includes “enjoy things” (item 20), “feel useful and needed” (item 17), “my life is pretty full” (item 18), “mind is clear as ever” (item 11), “easy to make decisions” (item 16), “hopeful about future” (item 14), “easy to do things” (item 12), “I enjoy attractive men/women” (item 6), and “feel best in morning” (item 2). Negative symptoms subscale includes “have crying spells” (item 3), “feel downhearted, sad, blue” (item 1), “more irritable than usual” (item 15), “restless and cannot keep still” (item 13), “tired for no reason” (item 10), “have trouble sleeping” (item 4), “heart beats faster than usual” (item 9), and “others better off if I were dead” (item 19). Somatic symptoms subscale includes “I am losing weight” (item 7), “eat as much as usual” (item 5), and “trouble with constipation” (item 8). 

We tried to create three subscales of the SDS by adding scores of the items belonging to each subscale. However, item 6 of the positive symptoms, items 4, 9, 15, and 19 of the negative symptoms, and item 7 of the somatic symptoms were reversely correlated with other item scores of each total score; thus, they were excluded from the summation to create the subscale scores.

In order to analyse the relationship of depression syndrome and temperament and character scales, we examined means, SDs, and internal consistency (measured as Cronbach's alpha coefficient) of all the variables used in this study. We then correlated all of them. We set alpha level at 0.001 rather than 0.05 because of multiple comparisons.

**Figure 1:**
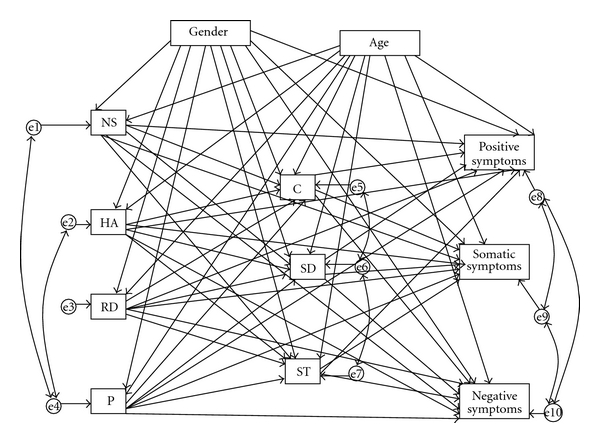
The original model. NA: negative affectivity; NS: novelty seeking; HA: harm avoidance; RD: reward dependence; P: persistence; SD: self-directedness; C: cooperativeness; ST: self-transcendence. The correlations among error variables in temperament/character dimensions were not indicated.

The associations between the depressive and personality scales were studied with the following hypotheses. Because Cloninger hypothesized that character domains would develop based on the temperament domain profiles, we posited that all the temperament scales would predict both the character domain scales and the depressive symptomatology scales. We also posited that the character domain scales would predict the depressive symptomatology scales. Both gender and age of the participant were expected to predict all the personality and depressive symptomatology scales. According to these hypotheses, we created a structural equation model (SEM) (Figure 1).

Statistical analyses were performed using SPSS 18.0 and AMOS 18.0 [22]. The fit of the CFA model was examined in terms of chi-squared (CMIN), goodness-of-fit index (GFI), adjusted goodness-of-fit index (AGFI), comparative fit index (CFI), and root mean square error of approximation (RMSEA). According to conventional criteria, a good fit would be indicated by CMIN/df < 2, GFI > 0.95, AGFI > 0.90, CFI > 0.95, and RMSEA < 0.05; an acceptable fit by CMIN/df < 3, GFI > 0.90, AGFI > 0.85, CFI > 0.85, and RMSEA < 0.10 [23].

## 3. Results

### 3.1. Characteristics of the TCI and SDS Subscales

Table 1 shows the means, SDs, and internal consistency of all the SDS and TCI scale scores. The Cronbach's alpha coefficients of the three SDS scales ranged between 0.44 and 0.80. Those of the seven TCI scales ranged from 0.41 to 0.81 for the temperament scales and from 0.65 to 0.82 for the character scales.

The correlations between the scales of TCI and SDS are also shown in Table 1. High HA and low SD were significantly correlated only with the positive symptom scores but, unexpectedly reversed with the negative as well as somatic symptoms scores. Among the SDS subscale scores, the positive symptoms scores were inversely correlated with the negative and somatic symptom scores whereas the latter two scores were positively correlated. Among the temperament subscales, NS and HA were inversely correlated with P whereas among the character subscales, SD was correlated positively with C and inversely with ST. Between temperament and character subscales, NS and HA were inversely correlated with SD and C; RD was correlated with C; P was correlated with SD, C, and ST.

### 3.2. The Relations between Personality and Depression in a SEM Path Analysis

We posited the original model with covariances between error variables of NS and HA with that of P as well as between error variables of C and ST with that of SD because of significant correlations observed in bivariate correlations. This model yielded CMIN/df = 1.8, GFI = 0.996, AGFI = 0.950, CFI = 0.996, and RMSEA = 0.042 (90% CI = 0.000–0.081). These indices suggested a good fit of the model with the data.

**Figure 2:**
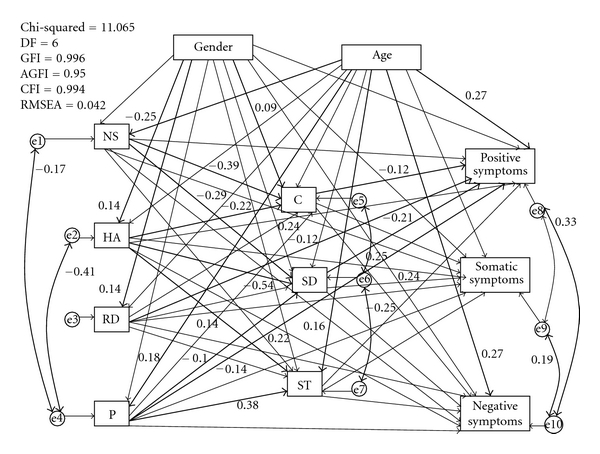
The path model with estimates. NA: negative affectivity; NS: novelty seeking; HA: harm avoidance; RD: reward dependence; P: persistence; SD: self-directedness; C: cooperativeness; ST: self-transcendence. All standardized parameter estimates in bold are significant (*P* < 0.05). Estimates without significance are in fine lines.

In this model (Figure 2), the positive symptoms scores were predicted by low C, SD, RD, and P as well as older age; the negative symptoms scores were predicted only by older age, and; the somatic symptoms scores were predicted by high SD. SD and C were predicted by low NS and low HA; C was predicted by RS as well as female gender; ST was predicted by high NS, HA, and P as well as older age.

## 4. Discussion

To the best of our knowledge, this study is the first to examine the differential associations of the TCI scales and different syndromes of depression. We also studied this issue taking the proposal of Cloninger into account that character develops based on temperament.

Depression has been thought of as compilation of different symptoms. There were many studies demonstrating several factors of depressive symptoms using a variety of rating instruments. And yet it has been not very common to examine the links of risky factors such as personality traits as in this study after dividing depressive symptoms into discrete syndromes. Our study showed the three depressive syndrome scores—the positive, negative, and somatic symptom scores—had unique links with the TCI subscale scores. 

High HA and low SD have usually been reported as associated with depression. However, our study showed that high HA and low SD were linked only with the positive symptoms scores in a bivariate analysis. This suggests that lack of positive mood (such as “enjoy things,” “feel best in morning”) and cognition (“feel useful and needed,” “my life is pretty full,” “mind is clear as ever,” “easy to make decisions,” “hopeful about future,” and “easy to do things”) were associated with this personality trait pattern.

HA is the temperament trait that many studies demonstrated connecting to depression [7, 9, 11, 12, 24–37]. Only a few studies showed contradictory results [38, 39]. However, high HA is not specific to depression. It was reported to be associated with panic disorder [40], social phobia [41], specific phobia [42], obsessional compulsive disorder [43–45], posttraumatic stress disorder [46], anorexia nervosa [47], bulimia nervosa [46, 48], somatization disorder [49], body dysmorphic disorder [50], schizophrenia [51], primary insomnia [52], pain [53], attention deficit/hyperactivity disorder [54, 55], autism spectrum disorders [54], and anxiety in general [12]. Hence, high HA may be a nonspecific trait for anxiety rather than depression *per se*. In this study, high HA was linked not to affective syndrome but to cognitive syndrome. Thus, high HA may be a risk factor of cognitive dysfunctioning that in turn makes individual vulnerable to anxiety (such as worrying, pessimism, shyness, and being fearful and doubtful [56]) of different types of psychopathology.

Another unique finding of this study is the lack of a direct link from high HA towards any of the depressive syndromal scores. HA predicted low SD and C that in turn predicted the positive symptoms scores. Thus, low SD and C mediated the effects of HA on the positive symptoms scores.

As in high HA, low SD was also known as a risk factor of depression [7, 9, 11, 12, 24–36]. Yet again, low SD is not a risky factor specific to depression. Low SD was reported to be associated with many other axis I and axis II disorders. In the present study, low SD was linked to lack of positive mood and cognition. Hence, low SD may be a nonspecific risky factor of psychological maladjustment.

Low RD and P were also reported in some studies as a risky factor of depression [12, 24, 27, 29, 34]. In this study, low RD and P were associated only with positive symptoms scores.

The positive symptoms scores were also linked to low C in this study. This was echoed in some previous studies [7, 9, 12, 26–29, 32, 34]. People low in affectionate ties with others may be more likely to feel depressed. Cooperativeness trait insists on coordination, harmony, solidarity, and so on. Low C may mean unsophisticatedness, being inconsonant, or even unsociable, and then can induce poor interpersonal relationship or low social support. Under these conditions, when an individual is hit by a crisis or suffers from a blow, without sufficient or effective social support or emotional platform, he or she may be in the lack of positive mood or cognition.

The uniqueness of the study is the examination of differential links of the TCI subscale scores with the three depressive syndromal scores. Most of the previous studies examined the association of the TCI subscale scores with the severity of depression as a whole. They rarely studied such association in different syndromes of depression. Our study suggested that while low SD, C, RD, and P predicted lack of positive mood and cognition, none of the TCI subscale scores except SD predicted the negative symptoms scores. Unexpectedly, *high* SD predicted the severity of the somatic symptoms scores after controlling the effects of all the other variables. This was what we did not expect and could not explain without difficulty. The negative symptoms scores (i.e., “have crying spells,” “feel downhearted, sad, blue,” “restless and cannot keep still,” “tired for no reason”) are thought of as core symptoms of depression and yet were predicted by none of the TCI subscales but by older age. Our study suggested different personality dimensions would predict different syndromes of depression.

Limitations of this study should be considered. This study was cross sectional. Hence the results may not indicate causality. Links posited in the path model were hypothetical and thus may be interpreted in reverse directions. Longitudinal studies following individuals with a set of measurements (e.g., [57]) may clarify the causality issue. Another drawback of this study was heavy reliance on self-report questionnaire. Depression may be better assessed by structured interviews. A third drawback is the fact that we used only nonclinical population. Studies on clinical populations may reveal different findings.

Although we relied on the meta-analysis of Shafer [20] of the SDS factor structure in order to make it easy to make international comparison, it remains to be further studied whether the factor structure of depression symptoms such as those measured by the SDS among a Chinese population would be the same as that reported in the Western countries. For example, the internal consistency was good for the positive symptoms score but fair or even worse for the other two SDS subscale scores. The factor structure of the SDS was reported using a Japanese population [19, 21], out of which Kitamura and colleagues' [58] reported the factor structure of the SDS in a fairly large (more than 20,000) population in Japan. An exploratory factor analysis yielded three factors—affective, cognitive, and somatic. Their affective factor included items such as “feel downhearted, sad, blue” (item 1), “have crying spells” (item 3), “heart beats faster than usual” (item 9), “tired for no reason” (item 10), “restless and cannot keep still” (item 13), “more irritable than usual” (item 15), and “others better off if I were dead” (item 19). Thus, this factor corresponds to Shafer's [20] negative symptoms. Kitamura et al.'s [58] cognitive factor included items such as “hopeful about future” (item 14), “easy to make decisions” (item 16), “feel useful and needed” (item 17), and “my life is pretty full” (item 18). Hence, this factor corresponded to Shafer's [20] positive symptoms. Kitamura et al.'s [58] Somatic factor included items such as “eat as much as usual” (item 5), “I enjoy attractive men/women” (item 6), and “easy to do things” (item 12). This factor differed from Shafer's [20] somatic symptoms. Therefore, the factor structures of the SDS in East Asian countries may not be very different from each other as well as from those in Western countries.

Another methodological concern of this study is relatively poor internal consistency of the TCI subscale scores. Cronbach's alpha was over 0.70 in HA, P, SD, and ST. Use of personality measures developed in the Western countries such as the TCI should be considered with caution when applying in a non-Western country like China. We used the Chinese version of the TCI which was one of the early versions of the measure. We should use the revised TCI (TCI-R) in a future study.

Finally, we should be very cautious about the robustness of the results. Ideally, we should solicit a larger and representative population in China. A resampling method such as bootstrapping may have to be considered. However, bootstrapping may potentially magnify the effects of unusual features in a data set and is not a magical means to compensate unrepresentativeness of the data [59, page 43]. 

Taking these methodological shortcomings into consideration, this study suggests that syndromes of depression are differentially associated with temperament and character patterns and that the effects of temperament on depression are mediated through character.

## Figures and Tables

**Table 1:** Correlations, means, SDs, and internal consistency of the SDS and TCI scores.

	(1)	(2)	(3)	(4)	(5)	(6)	(7)	(8)	(9)	(10)	(11)	(12)
(1) Positive symptoms	—											
(2) Negative symptoms	−0.48***	—										
(3) Somatic symptoms	−0.43***	0.63***	—									
(4) NS	0.09	−0.13**	−0.06	—								
(5) HA	0.19***	−0.39***	−0.35***	0.08	—							
(6) RD	−0.15**	0.04	0.00	0.07	−0.00	—						
(7) P	−0.13**	0.20***	0.12	−0.23***	−0.43***	0.03	—					
(8) SD	−0.30***	0.41***	0.36***	−0.32***	−0.51***	−0.02	0.20***	—				
(9) C	−0.24***	0.26***	0.14**	−0.42***	−0.26***	0.23***	0.24***	0.42***	—			
(10) ST	0.08	−0.07	−0.12*	0.11*	−0.02	0.08	0.30***	−0.23***	−0.03	—		
(11) Age	0.20***	0.12**	−0.04	−0.25***	−0.08	−0.05	0.19***	0.16***	0.16**	0.16***	—	
(12) Gender (men, 1; women, 2)	−0.06	0.05	−0.09*	−0.02	0.15**	0.15**	−0.11*	−0.01	0.10*	−0.01	−0.09*	—
Number of items	7	4	2	20	20	20	20	20	20	20	1	1
*M*	13.0	12.5	6.0	54.2	55.4	60.7	69.4	63.9	65.3	56.9	41.7	1.5
SD	3.9	2.4	1.2	8.0	9.4	6.8	10.4	9.1	8.3	11.4	11.9	0.5
Alpha	0.80	0.65	0.44	0.58	0.74	0.41	0.81	0.71	0.65	0.82	—	—

*Note*. NA: negative affectivity; NS: novelty seeking; HA: harm avoidance; RD: reward dependence; P: persistence; SD: self-directedness; C: cooperativeness; ST: self-transcendence.

*P < 0.05; **P < 0.01.
